# Comparison of the effectiveness of ranibizumab monotherapy vs. combined panretinal photocoagulation on the regression of neovascularization in proliferative diabetic retinopathy

**DOI:** 10.3389/fmed.2026.1808957

**Published:** 2026-04-28

**Authors:** Yuqing Wang, Lin Wang, Zhi Zheng, Jiajun Xu, Zhaode Zhang

**Affiliations:** 1 Department of Ophthalmology, Shanghai General Hospital, Shanghai Jiao Tong University School of Medicine, Shanghai, China; 2 Department of Ophthalmology, Ningde Clinical Medical College of Fujian Medical University, Ningde Municipal Hospital, Ningde, Fujian, China; 3 Eye Hospital, The First Hospital Affiliated of Harbin Medical University, Harbin, Heilongjiang, China

**Keywords:** diabetic retinopathy, neovascularization, photocoagulation, ranibizumab, treatment outcome

## Abstract

**Background:**

The optimal treatment strategy for proliferative diabetic retinopathy (PDR) remains an area of active investigation. This study aimed to compare the effectiveness of ranibizumab monotherapy vs. ranibizumab combined with panretinal photocoagulation (PRP) on neovascularization regression in PDR.

**Methods:**

This retrospective study analyzed 238 patients with PDR treated between January 2019 and December 2023. Patients were classified into a monotherapy group (*n* = 125, ranibizumab only) and a combined PRP group (*n* = 113, ranibizumab + PRP). Neovascularization regression was assessed via fundus fluorescein angiography before treatment and at 12, 24 months. Best-corrected visual acuity (BCVA) and central retinal thickness (CRT) were measured before treatment and at 3, 6, 12, and 24 months. Treatment-related indicators (injection numbers, rescue therapy rates) and adverse events were recorded.

**Results:**

At 24 months, the proportion of patients with residual neovascularization on the disc (NVD) was significantly lower in the combined PRP group (8.85%) than in the monotherapy group (23.20%, *P* = 0.003). No significant inter-group differences were found in BCVA or CRT at any time point (all *P* > 0.05). The combined PRP group required fewer ranibizumab injections (10.86 ± 1.72 vs. 15.23 ± 2.84, *P* < 0.001) and had lower rates of rescue PRP (2.65% vs. 21.60%, *P* < 0.001), pars plana vitrectomy (4.42% vs. 15.20%, *P* = 0.006), and vitreous hemorrhage (6.19% vs. 19.20%, *P* = 0.003). Multivariate analysis confirmed combined therapy as an independent protective factor against non-regression (OR = 0.054, *P* < 0.001).

**Conclusion:**

For PDR, ranibizumab combined with PRP was associated with greater NVD regression, reduced treatment burden, and a lower risk of sight-threatening complications compared with ranibizumab monotherapy, while visual and anatomical outcomes were comparable between the two groups.

## 1 Introduction

Diabetic retinopathy (DR) stands as the leading cause of preventable blindness among the working-age population globally, with its proliferative diabetic retinopathy stage (PDR) representing a severe, sight-threatening complication (1, 2). The pathogenesis of PDR is driven by chronic hyperglycemia-induced retinal ischemia, which triggers a pathological overexpression of vascular endothelial growth factor (VEGF) (3). This key cytokine promotes the growth of fragile, abnormal new blood vessels (neovascularization) on the retinal surface and optic disc. These neovascular structures are inherently unstable, prone to bleeding, and associated with subsequent fibrovascular proliferation, culminating in vitreous hemorrhage, tractional retinal detachment, and irreversible vision loss (4).

For decades, panretinal photocoagulation (PRP) has been the cornerstone treatment for PDR, aiming to ablate peripheral ischemic retina to downregulate VEGF production and induce regression of neovascularization (5, 6). While effective in reducing the risk of severe vision loss, PRP is a destructive procedure often accompanied by significant side effects (7). These include inevitable constriction of the visual field, potential exacerbation of diabetic macular edema (DME), and impaired dark adaptation and contrast sensitivity, which collectively can compromise patients' quality of vision even when central acuity is preserved (8, 9).

The advent of intravitreal anti-VEGF therapy, such as ranibizumab, has revolutionized the management of retinal vascular diseases by offering a targeted, pharmacologic approach (10). By directly neutralizing VEGF, these agents can rapidly induce regression of neovascularization and resolve associated macular edema (11). Consequently, anti-VEGF monotherapy has emerged as a promising alternative to PRP, with the potential to achieve therapeutic goals while avoiding the destructive sequelae of laser (12). However, concerns persist regarding the durability of its effect, the necessity for frequent, potentially indefinite injections to maintain quiescence, and the long-term risk of neovascular recurrence or fibrosis under a treat-and-extend regimen (13).

This evolving landscape has naturally led to the exploration of a combined strategy, integrating the immediate, potent anti-angiogenic effect of ranibizumab with the sustained, ablative effect of PRP. Theoretically, this combination could leverage the strengths of both modalities: ranibizumab could induce rapid neovascular regression and control edema, potentially allowing for a less intensive or delayed PRP protocol, while PRP could provide a more durable reduction in the ischemic drive, possibly lowering the long-term burden of anti-VEGF injections (14). Yet, the comparative long-term efficacy of ranibizumab monotherapy vs. its combination with PRP specifically on the regression of neovascularization in PDR, alongside associated treatment burdens and complication profiles, requires further elucidation through robust clinical data. Therefore, this study aims to retrospectively compare the effectiveness of ranibizumab monotherapy vs. ranibizumab combined with PRP on promoting neovascular regression and influencing clinical outcomes in patients with PDR over a 24-month period.

## 2 Materials and methods

### 2.1 Research subject

**Figure 1:**
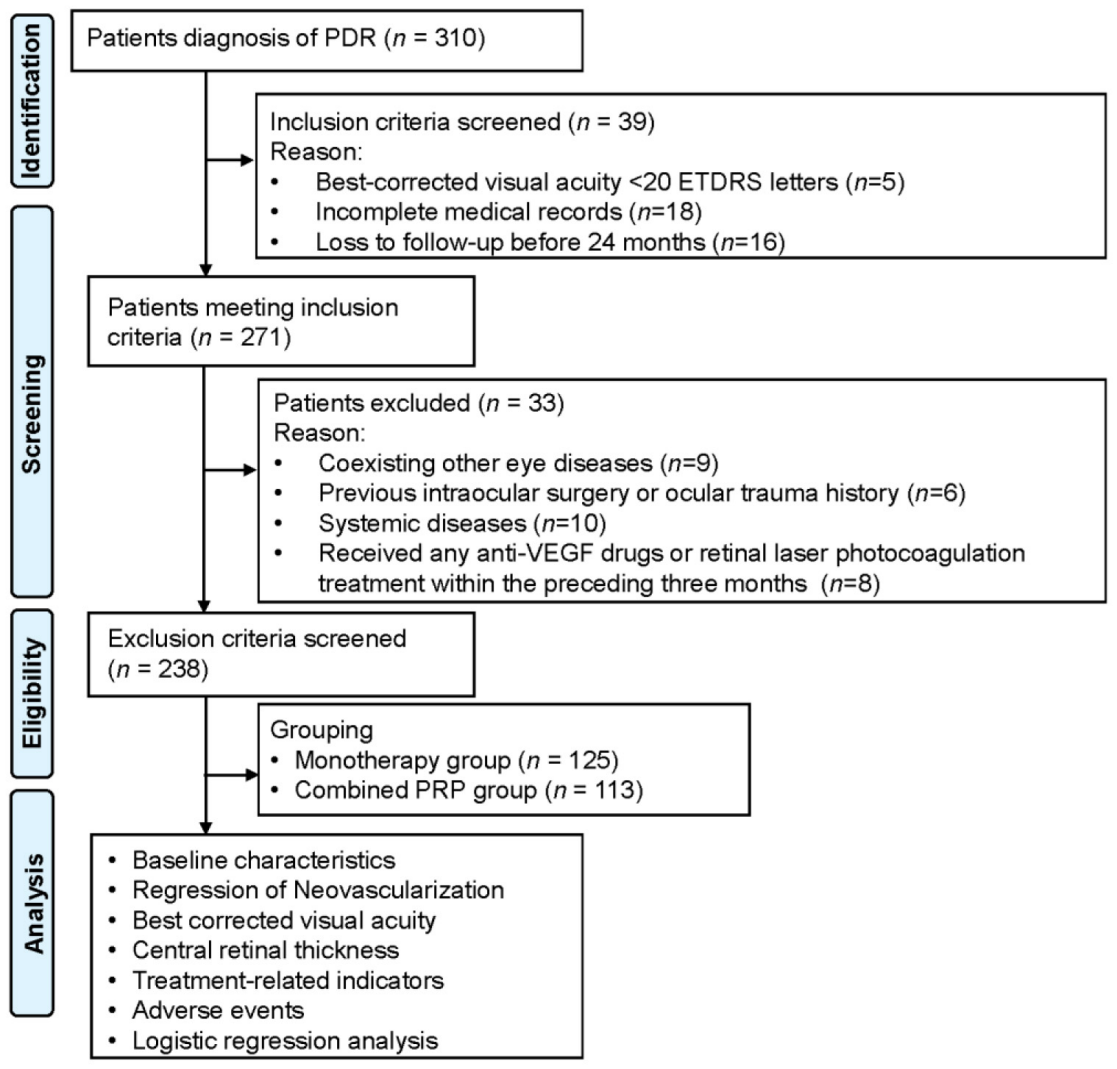
Patient flow diagram. PDR, proliferative diabetic retinopathy; anti-VEGF, anti-vascular endothelial growth factor; PRP, panretinal photocoagulation.

This study is a retrospective analysis involving 238 patients diagnosed and treated for proliferative diabetic retinopathy (PDR) at our hospital's ophthalmology department between January 2019 and December 2023 (Figure 1). The inclusion criteria were as follows: ① Diagnosis confirmed according to the “Guidelines for the Diagnosis and Treatment of Diabetic Retinopathy” via fundus fluorescein angiography (FFA) and spectral-domain optical coherence tomography (SD-OCT), showing retinal neovascularization (including neovascularization on the disc [NVD]/ neovascularization elsewhere [NVE]), and clinically significant diabetic macular edema (DME); ② Age ≥18 years; ③ Best-corrected visual acuity (BCVA) ≥20 Early Treatment Diabetic Retinopathy Study (ETDRS) letters (Snellen equivalent 20/400), with glycated hemoglobin (HbA1c) ≤ 12%; ④ For patients with bilateral PDR, the eye with more severe neovascularization was selected, or, if severity was comparable, the eye that received treatment first was chosen; ⑤ Complete medical records and completion of at least 24 months of regular follow-up. Exclusion criteria were as follows: ① Presence of significant media opacities that severely affect fundus observation or visual acuity assessment; ② Coexisting retinal vascular diseases, macular ischemia, active ocular inflammation or infection, glaucomatous optic neuropathy, or high myopia; ③ History of previous intraocular surgery or ocular trauma; ④ Uncontrolled hypertension, severe liver or renal insufficiency, malignant tumors, immunodeficiency disorders, or other systemic diseases; ⑤ Previous vitrectomy, or treatment with any anti-vascular endothelial growth factor (anti-VEGF) drugs or retinal laser photocoagulation for PDR within the preceding 3 months.

Based on the treatment regimens received, the 238 patients were defined into the monotherapy group (*n* = 125) and the combined panretinal photocoagulation (PRP) group (*n* = 113). The monotherapy group was defined as receiving only intravitreal ranibizumab injections. The combined PRP group was defined as receiving intravitreal ranibizumab injections along with PRP treatment.

### 2.2 Ethical statement

This study strictly adheres to the Declaration of Helsinki and relevant ethical guidelines for medical research, and has received formal approval from the Medical Ethics Committee of Shanghai General Hospital. According to the “Ethical Review Measures for Biomedical Research Involving Human Subjects” regarding the ethical requirements for retrospective studies, and considering the characteristics of this study—namely, no direct intervention, low risk, and adequate privacy protection—the Medical Ethics Committee reviewed and unanimously agreed to waive the informed consent process for this study.

### 2.3 Treatment regimens

(1) Ranibizumab treatment: Three days prior to treatment, patients were administered levofloxacin eye drops (Approval No. H20173405, Suzhou Lezhu Pharmaceutical Co., Ltd., Jiangsu Province). A needle was inserted vertically into the scleral surface 3.5 mm posterior to the temporal limbus, and 0.5 mg/0.05 mL of ranibizumab (Approval No. SJ20170003, Novartis Pharma Schweiz AG, Switzerland) was injected into the vitreous cavity. After needle withdrawal, pressure was applied with a cotton swab for 2 to 3 min, followed by application of tobramycin and dexamethasone ophthalmic ointment (Approval No. H20020496, Qilu Pharmaceutical Co., Ltd., Shandong Province). The treated eye was then bandaged, and levofloxacin eye drops were administered for 3 days. This treatment was repeated monthly for at least 3 consecutive months.

(2) PRP treatment: Three days prior to treatment, patients were routinely administered levofloxacin eye drops four times daily. Before the procedure, compound tropicamide eye drops (Approval No. H20083812, Heilongjiang Longgui Pharmaceutical Co., Ltd., Heilongjiang Province) were used for mydriasis, and 0.4% oxybuprocaine hydrochloride eye drops (Approval No. H20056587, Shandong Bausch & Lomb Freda Pharmaceutical Co., Ltd., Shandong Province) were used for topical anesthesia. A wide-angle fundus contact lens was placed on the cornea, avoiding the macular papillary bundle. The 532 nm argon green laser “C”-shaped grid-like multiple-point laser mode (LIGHTLAS 532, Quantel Medical, France) was employed. Photocoagulation was performed at a distance of 500 μm from the center of the macula, with a spot diameter (R) of 100 μm, exposure time of 0.05 s, laser energy of 100 mW, and spacing between adjacent spots of one spot width. The Tso intensity grade of the spots was I–II. Subsequently, the spot R was adjusted to 200–300 μm, exposure time to 0.05–0.20 s, laser energy to 120–300 mW, with spacing between adjacent spots of one spot width, and the Tso intensity grade remained at I–II. Disseminated photocoagulation was performed from outside the vascular arcades to the peripheral areas of the four quadrants. Postoperatively, levofloxacin eye drops were continued for 3 days, four times daily. The treatment was completed in two sessions, with a 1-week interval between each session. Additionally, patients in the combination PRP group received intravitreal injections of 0.5 mg/0.05 mL ranibizumab starting 1 week after their second PRP treatment, continuing for at least 3 months.

(3) Follow up and subsequent treatment adjustments: All patients underwent regular monthly monitoring for 24 months after the initiation of treatment. At each visit, all patients received BCVA measurements and SD-OCT assessments. FFA was performed every 3 months to document the regression of neovascularization. Re-treatment with ranibizumab was administered if any of the following criteria were met: A decrease in BCVA of ≥5 ETDRS letters (or ≥1 line on the Snellen chart) compared to the previous follow-up; Central retinal thickness (CRT) ≥320 μm; Worsening of neovascularization (NVD/NVE), indicated by an increase in leakage area or the appearance of new neovascular branches. If neovascularization persisted without regression over three consecutive follow-ups or showed signs of fibrovascular proliferation, rescue PRP was performed. Additionally, if a patient experienced persistent vitreous hemorrhage (obscuring the fundus, preventing completion of FFA and laser treatment) lasting more than 1 month without absorption or tractional retinal detachment involving the macula or extending beyond one quadrant, and conservative management was ineffective, pars plana vitrectomy (PPV) was performed.

### 2.4 Data collection

(1) Baseline characteristics: Baseline characteristics of all patients prior to treatment were collected through the medical record system. Demographic characteristics primarily included age, gender, body mass index (BMI), and comorbidities (hypertension, hyperlipidemia). Clinical characteristics mainly comprised diabetes mellitus (DM) type, duration of DM, DM treatment regimen, HbA1c levels, severity of PDR, and ellipsoid zone data. HbA1c levels were measured using high-performance liquid chromatography (Bio-Rad D-100, Bio-Rad Laboratories, Hercules, California (CA), USA). The status of the ellipsoid zone was determined using SD-OCT images (SPECTRALIS HRA+OCT, Heidelberg Engineering GmbH, Germany). The severity of PDR was classified as “high-risk” and “non-high-risk” according to the ETDRS criteria. “High-risk PDR” was defined as the presence of any one of the following conditions: (a) NVD area ≥ one-third of the standard disc area, or (b) NVD at any location accompanied by vitreous or preretinal hemorrhage, or (c) NVE area ≥ one-half of the standard disc area accompanied by vitreous or preretinal hemorrhage.

(2) Regression of neovascularization: Before treatment, and at 12 and 24 months post-treatment, widefield fluorescein angiography was performed using the SPECTRALIS HRA+OCT system (Heidelberg Engineering GmbH, Germany) with the widefield module (30° × 30° to 102° fields), allowing visualization of the peripheral retina. Full-retina montage images were constructed to evaluate neovascularization extent, particularly NVE. For patients in the combined PRP group, the 12-month assessment was performed at least 11 months after the final PRP session to minimize interference from transient post-laser hyperfluorescence. All FFA images were independently graded by the same retinal specialist who was masked to the patients' treatment allocation and all other clinical data. Regression of neovascularization was defined as the complete disappearance of the original neovascular structure with no evidence of fluorescein leakage. “No leakage” was operationally defined as the absence of hyperfluorescent borders or progressive dye accumulation beyond the early phase (30–60 s) on the FFA sequence, with confirmation by comparison to baseline images to differentiate residual neovascularization from background choroidal fluorescence or window defects.

(3) BCVA: Before treatment and at 3, 6, 12, and 24 months post-treatment, BCVA was assessed using the ETDRS chart under standard illumination conditions by the same professional optometrist. The number of ETDRS letters (range: 0–100 letters) obtained after optimal correction was recorded.

(4) CRT: Before treatment and at 3, 6, 12, and 24 months post-treatment, CRT was measured using a SD-OCT device (SPECTRALIS HRA+OCT, Heidelberg Engineering GmbH, Germany). Scans were centered on the fovea, and CRT (μm) was measured. The average of three consecutive scans was taken as the final result.

(5) Treatment-related indicators: During the follow-up period, the total number of intravitreal ranibizumab injections received by patients (including both initial treatment and re-treatments as needed) was recorded. Additionally, the rescue PRP rate (the proportion of patients in both groups requiring rescue PRP treatment due to persistent neovascularization) and the PPV rate (the proportion of patients in both groups requiring PPV treatment due to severe complications) were calculated.

(6) Adverse events: The main adverse events recorded during the follow-up period included vitreous hemorrhage (confirmed by fundus examination or B-scan ultrasonography), retinal detachment (diagnosed by fundus examination and SD-OCT), elevated intraocular pressure (intraocular pressure ≥ 25 mmHg, confirmed by multiple measurements to exclude physiological fluctuations), and cataract (lens opacity worsening compared to pre-treatment, affecting vision or hindering fundus observation).

### 2.5 Statistical analysis

This study used SPSS 29.0 statistical software (IBM Corp., Armonk, NY, USA) for data processing and analysis. All statistical tests were two-tailed, with *P* < 0.05 considered statistically significant. Normality of continuous variables was assessed using the Shapiro-Wilk test. The test results showed that the Shapiro-Wilk test *P*-*values* for all variables were greater than 0.05, supporting the assumption of normal distribution. Therefore, continuous variables are presented as means ± standard deviations, and group comparisons were performed using independent samples *t*-tests. For longitudinal outcomes (BCVA and CRT), linear mixed-effects models were used to evaluate between-group differences over time. The models included fixed effects for treatment group, time (baseline, 3, 6, 12, and 24 months), and the group-by-time interaction, with a random intercept for each patient to account for within-subject correlation. Between-group comparisons at each time point were derived from the model using estimated marginal means. Independent samples *t*-tests at individual time points were also performed for descriptive consistency; results were consistent with the mixed-model analyses. Categorical variables are expressed as frequencies and percentages [*n* (%)] and were compared between groups using the χ^2^ test. Additionally, binary logistic regression analyses (univariate and multivariate) were conducted with “non-regression of neovascularization in PDR at 24 months post-treatment” as the dependent variable (non-regression = 1, regression = 0) to identify independent risk or protective factors affecting the non-regression of neovascularization in PDR.

All analyses were performed based on the initial treatment group assignment (intention-to-treat principle). Patients who received rescue PRP or PPV during follow-up remained in their originally assigned group for the analysis of neovascularization regression, BCVA, and CRT. The rates of rescue PRP and PPV were analyzed as separate secondary outcomes to reflect treatment burden and disease control under each initial strategy.

## 3 Results

### 3.1 Baseline characteristics

A total of 238 patients diagnosed with PDR were included, comprising 125 patients in the ranibizumab monotherapy group and 113 patients in the combined PRP group. As shown in Table 1, there were no statistically significant differences between the two groups in terms of demographic characteristics (age, gender, BMI, hypertension, hyperlipidemia) or clinical characteristics (type of diabetes, duration of diabetes, treatment regimen for diabetes, HbA1c level, severity of PDR, or ellipsoid zone status) at baseline (all *P* > 0.05). This indicates that the two groups were comparable (Table 1).

**Table 1:** Comparison of demographic and clinical characteristics between two groups.

Parameter	Monotherapy group (*n* = 125)	Combined PRP group (*n* = 113)	*t/χ^2^*	*P*
Demographic				
Age (years)	62.14 ± 6.65	63.07 ± 7.21	1.037	0.301
Gender [*n* (%)]			0.064	0.800
Male	61 (48.80%)	57 (50.44%)		
Female	64 (51.20%)	56 (49.56%)		
BMI (kg/m^2^)	29.53 ± 6.72	30.48 ± 5.16	1.227	0.221
Hypertension [*n* (%)]	81 (64.80%)	74 (65.49%)	0.012	0.912
Hyperlipidemia [*n* (%)]	70 (56.00%)	65 (57.52%)	0.056	0.813
Clinical				
Type of DM [*n* (%)]			0.003	0.958
Type 1	38 (30.40%)	34 (30.09%)		
Type 2	87 (69.60%)	79 (69.91%)		
Duration of DM (years)	21.64 ± 6.85	22.87 ± 6.12	1.457	0.146
Treatment regimen of DM [*n* (%)]			0.296	0.587
Insulin	86 (68.80%)	74 (65.49%)		
No insulin	39 (31.20%)	39 (34.51%)		
HbA1c level (%)	7.88 ± 1.92	7.95 ± 1.46	0.342	0.733
Severity of PDR [*n* (%)]			0.208	0.648
Non-high-risk	59 (47.20%)	50 (44.25%)		
High-risk	66 (52.80%)	63 (55.75%)		
Ellipsoid zone [*n* (%)]			0.178	0.673
Intact	84 (67.20%)	73 (64.60%)		
Disrupted	41 (32.80%)	40 (35.40%)		

PRP, panretinal photocoagulation; BMI, body mass index; DM, diabetes mellitus; HbA1c, glycated hemoglobin; PDR, proliferative diabetic retinopathy.

### 3.2 Regression of neovascularization

The regression of NVD and NVE in both groups at different time points is presented in Table 2. Before treatment, the proportions of patients with NVD and NVE were similar between the monotherapy and combined PRP groups (*P* > 0.05). At 12 months post-treatment, the proportion of patients with residual NVD was significantly lower in the combined PRP group compared to the monotherapy group (15.93% vs. 32.00%, *P* = 0.004). This significant difference persisted at the 24-month follow-up (8.85% vs. 23.20%, *P* = 0.003). Regarding NVE, while the proportion of patients with residual NVE was lower in the combined PRP group at both 12 months and 24 months, these differences did not reach statistical significance (*P* = 0.097 and *P* = 0.095, respectively; Table 2).

**Table 2:** Comparison of the effect of neovascularization regression between two groups [*n* (%)].

Parameter	Monotherapy group (*n* = 125)	Combined PRP group (*n* = 113)	*χ^2^*	*P*
NVD				
Before treatment	82 (65.60%)	76 (67.26%)	0.073	0.787
12 months after treatment	40 (32.00%)	18 (15.93%)	8.316	0.004
24 months after treatment	29 (23.20%)	10 (8.85%)	8.920	0.003
NVE				
Before treatment	65 (52.00%)	52 (46.02%)	0.850	0.357
12 months after treatment	20 (16.00%)	10 (8.85%)	2.755	0.097
24 months after treatment	11 (8.80%)	4 (3.54%)	2.781	0.095

PRP, panretinal photocoagulation; NVD, neovascularization on the disc; NVE, neovascularization elsewhere.

### 3.3 Best corrected visual acuity

The changes in BCVA (measured in ETDRS letters) for both groups are shown in Table 3. At baseline, BCVA was comparable between the two groups. BCVA improved significantly in both groups over the 24-month follow-up period. Linear mixed-effects modeling revealed no significant main effect of treatment group (*F* = 1.62, *P* = 0.204) and no significant group-by-time interaction (*F* = 0.71, *P* = 0.585), indicating that BCVA trajectories did not differ between the monotherapy and combined PRP groups. Pairwise comparisons at individual time points derived from the model confirmed no statistically significant differences between groups at any post-treatment visit (all *P* > 0.05; Table 3).

**Table 3:** Comparison of the BCVA based on linear mixed effects model (letters).

Parameter	Monotherapy group (*n* = 125)	Combined PRP group (*n* = 113)	Between-group difference (95% CI)	*P*
Before treatment	53.85 ± 7.24	54.12 ± 6.95	−0.27 (−1.95, 1.41)	0.768
3 months after treatment	56.41 ± 8.13	57.58 ± 7.40	−1.17 (−2.96, 0.62)	0.199
6 months after treatment	60.37 ± 8.49	60.92 ± 8.06	−0.55 (−2.42, 1.32)	0.563
12 months after treatment	69.75 ± 8.65	70.94 ± 8.03	−1.19 (−3.03, 0.65)	0.205
24 months after treatment	69.13 ± 9.11	71.26 ± 7.92	−2.13 (−4.31, 0.05)	0.056

The model showed no significant main effect of treatment group (F = 1.62, P = 0.204) and no significant group-by-time interaction (F = 0.71, P = 0.585).

PRP, panretinal photocoagulation; BCVA, best corrected visual acuity; CI, confidence interval.

### 3.4 Central retinal thickness

The changes in CRT for both groups are summarized in Table 4. Baseline CRT was similar between the groups. CRT decreased significantly in both groups following treatment. Linear mixed-effects modeling showed no significant main effect of treatment group (*F* = 1.15, *P* = 0.285) and no significant group-by-time interaction (*F* = 0.94, *P* = 0.442), indicating that CRT changes over time did not differ between the two groups. Pairwise comparisons derived from the model confirmed no statistically significant differences at any post-treatment visit (all *P* > 0.05; Table 4).

**Table 4:** Comparison of the CRT based on linear mixed effects model (μm).

Parameter	Monotherapy group (*n* = 125)	Combined PRP group (*n* = 113)	Between-group difference (95% CI)	*P*
Before treatment	482.36 ± 68.75	475.84 ± 71.62	6.52 (−10.92, 23.96)	0.475
3 months after treatment	382.18 ± 48.26	391.47 ± 52.33	−9.29 (−21.78, 3.20)	0.144
6 months after treatment	351.42 ± 38.14	356.89 ± 41.05	−5.47 (−15.83, 4.89)	0.300
12 months after treatment	327.03 ± 33.59	332.76 ± 34.12	−5.73 (−14.56, 3.10)	0.202
24 months after treatment	315.21 ± 35.84	321.53 ± 32.67	−6.32 (−15.43, 2.79)	0.174

The model showed no significant main effect of treatment group (F = 1.15, P = 0.285) and no significant group-by-time interaction (F = 0.94, P = 0.442).

PRP, panretinal photocoagulation; CRT, central retinal thickness; CI, confidence interval.

### 3.5 Treatment-related indicators

**Figure 2:**
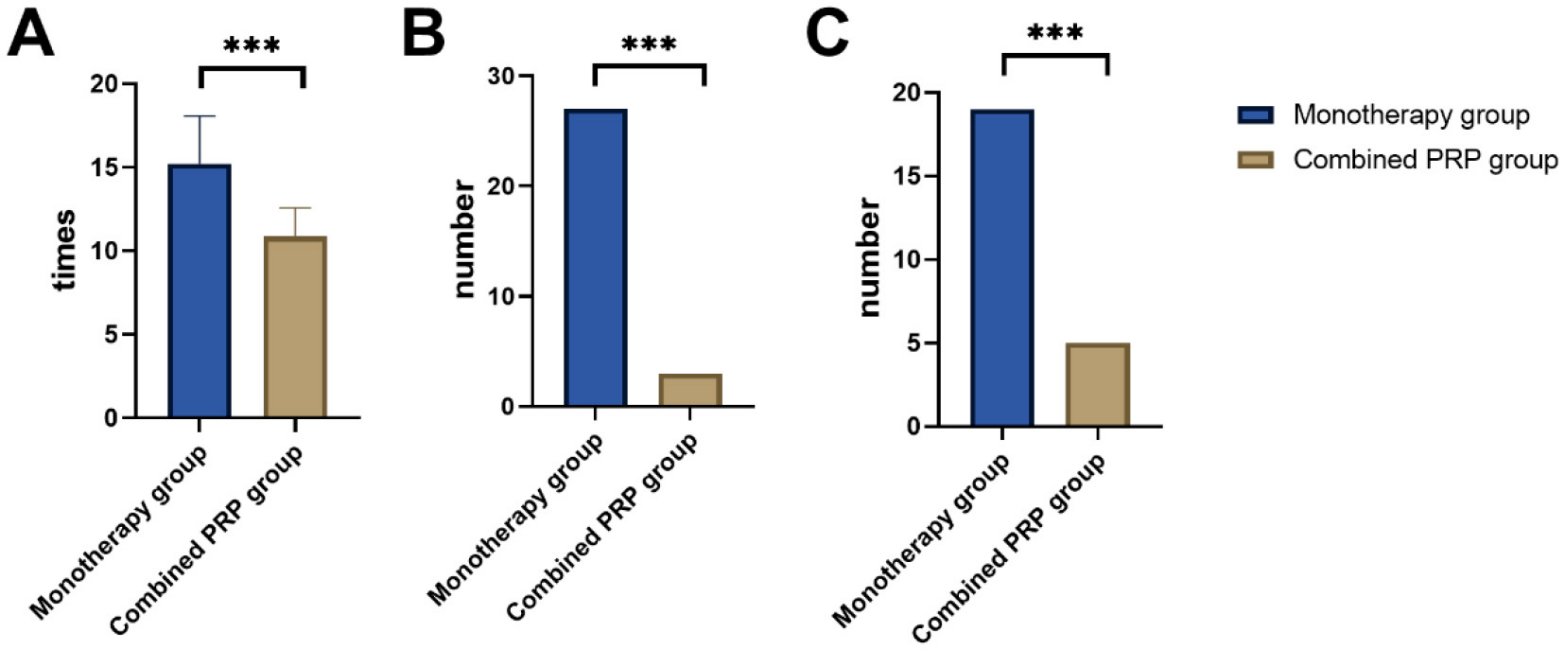
Comparison of the treatment-related indicators between two groups. **(A)** Total number of ranibizumab injections (times); **(B)** Rescue PRP (*n*); **(C)** PPV (*n*). PRP, panretinal photocoagulation; PPV, pars plana vitrectomy. ***P < 0.001.

Treatment-related indicators are compared in Figure 2. The total number of ranibizumab injections received during the 24-month follow-up was significantly lower in the combined PRP group compared to the monotherapy group (10.86 ± 1.72 vs. 15.23 ± 2.84, *P* < 0.001). The rescue PRP rate was significantly higher in the monotherapy group (21.60% vs. 2.65%, *P* < 0.001). Similarly, the PPV rate was significantly higher in the monotherapy group (15.20% vs. 4.42%, *P* = 0.006; Figure 2).

### 3.6 Adverse events

**Figure 3:**
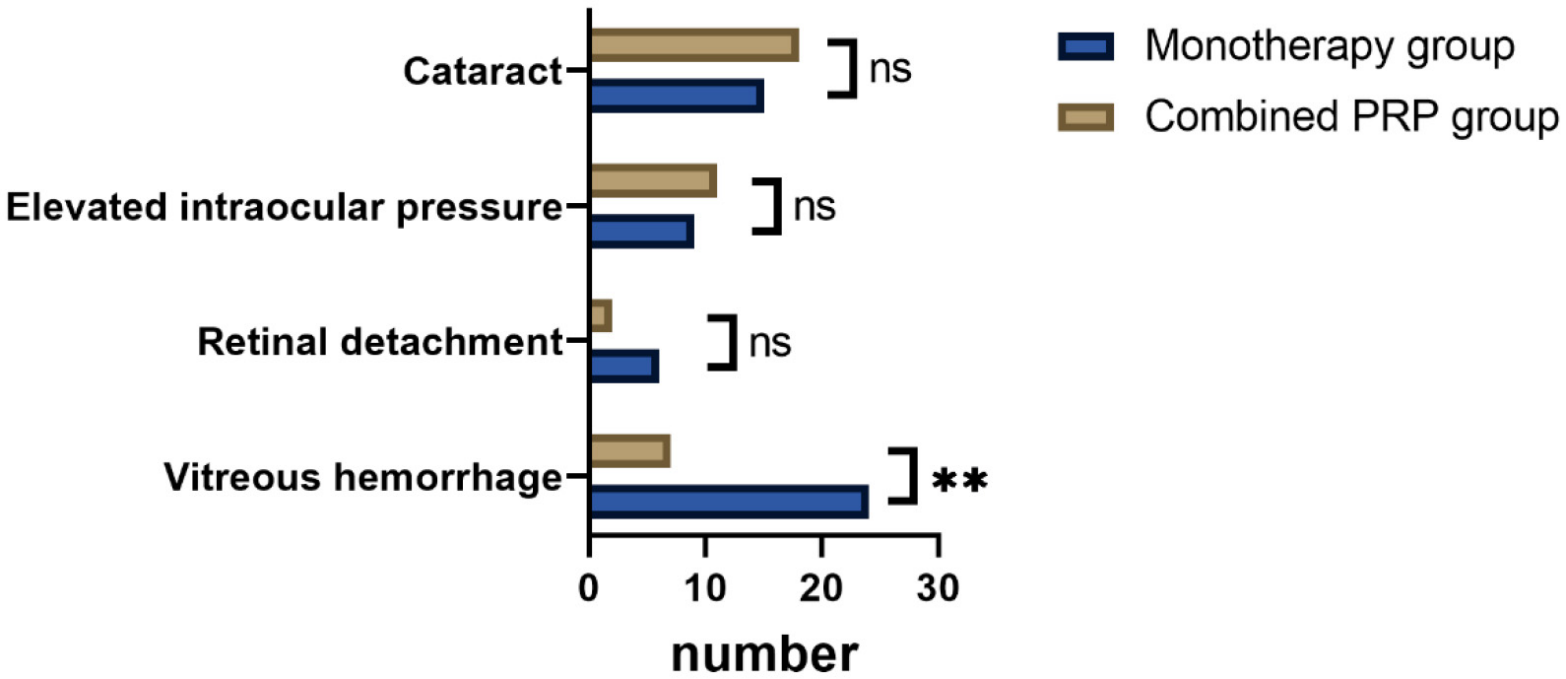
Comparison of adverse events between two groups (*n*). PRP, panretinal photocoagulation. ns, no statistically significant difference; **P < 0.01.

The incidence of adverse events during the follow-up period is detailed in Figure 3. The incidence of vitreous hemorrhage was significantly higher in the monotherapy group compared to the combined PRP group (19.20% vs. 6.19%, *P* = 0.003). There were no statistically significant differences between the two groups in the incidence of retinal detachment (*P* = 0.350), elevated intraocular pressure (*P* = 0.482), or cataract progression (*P* = 0.381; Figure 3).

### 3.7 Univariate and multivariate logistic regression analysis

Univariate logistic regression analysis identified several factors associated with the non-regression of neovascularization at 24 months. Ranibizumab combined with PRP was a significant protective factor (OR = 0.058, 95% CI 0.022–0.156, *P* < 0.001). Longer duration of diabetes (OR = 1.093, 95% CI 1.036–1.153, *P* = 0.001), higher HbA1c level (OR = 1.425, 95% CI 1.144–1.775, *P* = 0.002), high-risk PDR status (OR = 2.057, 95% CI 1.092–3.877, *P* = 0.026), and a disrupted ellipsoid zone (OR = 1.984, 95% CI 1.079–3.650, *P* = 0.028) were identified as risk factors (Table 5).

**Table 5:** Univariate logistic regression analysis of risk factors for the non-regression of neovascularization in PDR.

Parameter	Coefficient	Std error	Wald	*P*	OR	95%CI
Ranibizumab combined PRP	−2.847	0.502	32.189	< 0.001	0.058	0.022–0.156
Duration of DM	0.089	0.027	10.856	0.001	1.093	1.036–1.153
HbA1c	0.354	0.112	9.992	0.002	1.425	1.144–1.775
High-risk PDR	0.721	0.323	4.985	0.026	2.057	1.092–3.877
Disrupted ellipsoid zone	0.685	0.311	4.850	0.028	1.984	1.079–3.650

PDR, proliferative diabetic retinopathy; PRP, panretinal photocoagulation; OR, odds ratio; CI, confidence interval; DM, diabetes mellitus; HbA1c, glycated hemoglobin.

Multivariate logistic regression analysis was performed including all significant factors from the univariate analysis. In the final model, ranibizumab combined with PRP remained a strong independent protective factor (OR = 0.054, 95% CI 0.020–0.148, *P* < 0.001). Longer duration of diabetes (OR = 1.079, 95% CI 1.022–1.140, *P* = 0.007) and higher HbA1c level (OR = 1.388, 95% CI 1.108–1.740, *P* = 0.004) remained significant independent risk factors for non-regression. High-risk PDR status (OR = 1.906, 95% CI 0.997–3.646, *P* = 0.051) and disrupted ellipsoid zone (OR=1.818, 95% CI 0.955–3.462, *P* = 0.069) showed a trend toward being risk factors but were no longer statistically significant in the multivariate model (Table 6).

**Table 6:** Multivariate logistic regression analysis of risk factors for the non-regression of neovascularization in PDR.

Parameter	Coefficient	Std error	Wald stat	*P*	OR	OR CI lower	OR CI upper
Ranibizumab combined PRP	−2.912	0.512	32.359	< 0.001	0.054	0.020	0.148
Duration of DM	0.076	0.028	7.372	0.007	1.079	1.022	1.140
HbA1c	0.328	0.115	8.126	0.004	1.388	1.108	1.740
High-risk PDR	0.645	0.331	3.799	0.051	1.906	0.997	3.646
Disrupted ellipsoid zone	0.598	0.329	3.306	0.069	1.818	0.955	3.462

PDR, proliferative diabetic retinopathy; PRP, panretinal photocoagulation; OR, odds ratio; CI, confidence interval; DM, diabetes mellitus; HbA1c, glycated hemoglobin.

## 4 Discussion

This retrospective study provides a comparative analysis of two contemporary management strategies for PDR over a 24-month period. The core findings indicate that while both ranibizumab monotherapy and combination therapy with PRP were associated with improvements in visual and anatomical parameters, the combined approach was associated with greater neovascular regression, a lower long-term treatment burden, and a reduced risk of serious complications in this cohort. These observations contribute to the ongoing debate regarding the optimal initial therapeutic strategy for PDR.

The most salient difference between the two groups was observed in the regression of neovascularization, particularly NVD. Patients receiving combination therapy exhibited a substantially higher rate of complete NVD regression compared to those on monotherapy at both the 12- and 24-month endpoints. This aligns with the fundamental pathophysiological rationale for combining anti-VEGF agents with PRP (15). While intravitreal ranibizumab provides rapid, potent pharmacological inhibition of VEGF, its effect is transient. PRP, by contrast, achieves a more sustained reduction in the overall retinal ischemic drive, which is the primary stimulus for VEGF upregulation (16). The combination thus appears to offer a synergistic or at least additive effect: ranibizumab induces an immediate shutdown of active neovascular complexes, potentially reducing the risk of early hemorrhage, while PRP addresses the underlying metabolic demand, promoting a more durable and complete involution of new vessels (17, 18). The differential effect on NVD vs. NVE is noteworthy and may reflect the distinct angiogenic microenvironments; NVD, often associated with more severe global ischemia, might be more dependent on the broad-scale ischemic modulation provided by PRP (19).

The enhanced neovascular control in the combination group translated directly into meaningful differences in treatment intensity and need for rescue interventions. The total number of ranibizumab injections required over 2 years was markedly lower in the combination therapy group. This represents a significant “treatment-modifying effect” of PRP, whereby the initial laser treatment reduces the subsequent dependency on repeated anti-VEGF injections to maintain quiescence (20). This finding is consistent with several previous trials, such as the DRCR.net Protocol S, which reported a similar reduction in anti-VEGF injection frequency with combined or delayed laser approaches compared to monotherapy (21). Furthermore, the rates of both rescue PRP and eventual pars plana vitrectomy were considerably higher in the monotherapy group. These data suggest that a proportion of eyes managed with anti-VEGF alone may experience an incomplete or unstable response, characterized by persistent or recurrent neovascular activity that necessitates escalation to laser or surgery. The combination strategy, therefore, may serve as a more definitive first-line intervention, potentially altering the natural history of the disease and reducing the likelihood of progressing to surgical endpoints (22, 23).

The complication profile further underscores the potential benefits of a combined approach. The incidence of vitreous hemorrhage was notably higher in the monotherapy cohort. This can be plausibly explained by the mechanism of action. Anti-VEGF agents cause rapid contraction and involution of neovascular fronds but may not always lead to complete fibrosis and obliteration of the vascular channel (24, 25). If treatment intervals are extended or if there is a rebound in VEGF levels, these fragile vessels may recanalize and bleed (26, 27). PRP, by promoting atrophy and fibrosis of the feeder vessels and the ischemic retina, may lead to a more structurally stable regression, thereby offering better protection against spontaneous vitreous hemorrhage, a common and vision-threatening event in PDR (28).

Our regression analysis corroborated these clinical observations, identifying combination therapy as a strong independent protective factor against the failure of neovascular regression at 24 months. Conversely, a longer duration of diabetes and higher baseline HbA1c levels were confirmed as independent risk factors. This reinforces the critical importance of systemic glycemic control in modulating local ocular disease activity and treatment response (29, 30). Even with advanced ocular interventions, poor systemic metabolic parameters can undermine therapeutic efficacy, highlighting the necessity for multidisciplinary management. The trend observed for high-risk PDR status and a disrupted ellipsoid zone suggests that more severe baseline disease may also portend a less favorable response, although these factors were not independent in our final model.

This study has several limitations inherent to its retrospective design. First, treatment allocation was not randomized, potentially introducing selection bias, although the baseline characteristics were well-balanced. The follow-up and re-treatment protocols, while standardized in description, may have been subject to individual clinician discretion in practice. The single-center nature of the study may limit the generalizability of the findings. Second, we did not assess patient-reported outcomes or functional measures such as visual field and contrast sensitivity, which are relevant for evaluating the full impact of PRP. Third, the inclusion criteria in this study required patients to have clinically significant DME in addition to PDR, with a baseline BCVA of at least 20 ETDRS letters and HbA1c ≤ 12%. Consequently, the study cohort represents a specific subset of PDR patients—those with concomitant DME and without extremely poor vision or severely uncontrolled systemic glycemia. The findings may therefore not be directly generalizable to PDR patients without DME, those presenting with very low vision, or individuals with HbA1c levels exceeding 12%. Future studies should investigate whether similar comparative outcomes are observed in broader PDR populations. Fourth, the treatment protocols differed not only in the inclusion of PRP but also in the sequence and timing of interventions. In the combined group, PRP was completed before initiating ranibizumab, whereas the monotherapy group received ranibizumab alone from the outset. This design reflected the clinical practice at our institution during the study period, where PRP was often prioritized early in the combined approach to achieve durable ischemia reduction. The earlier application of PRP in the combined group may have contributed to the observed lower retreatment rates and more favorable neovascular regression, independent of the presence of PRP itself. Therefore, the results should be interpreted with the understanding that the timing of PRP relative to anti-VEGF initiation may have influenced outcomes, and future randomized studies with standardized timing protocols are needed to isolate the true effect of combining modalities. Fifth, a relatively high proportion of patients in the monotherapy group received rescue PRP due to insufficient efficacy, which effectively altered their treatment exposure. Our primary analysis is based on the initial treatment assignment, which may underestimate the limitations of anti-VEGF monotherapy to some extent and may overestimate the differences in outcomes between initial combination strategies and monotherapy under “pure exposure” conditions. Although this reflects real-world clinical practice (i.e., treatment escalation), this factor should still be considered when interpreting differences in outcomes such as neovascular regression between the two groups.

## 5 Conclusion

In conclusion, within this retrospective cohort of patients with PDR and concomitant DME, combination therapy with intravitreal ranibizumab and PRP was associated with higher rates of regression of NVD, a lower treatment burden, and fewer sight-threatening complications compared with ranibizumab monotherapy over 24 months, while regression of NVE did not differ significantly between groups. Visual and anatomical outcomes were similar between the two treatment strategies. These findings suggest that the addition of PRP to anti-VEGF therapy may offer advantages in disease control, although prospective randomized studies are needed to confirm these associations and establish causality.

## Data Availability

The raw data supporting the conclusions of this article will be made available by the authors, without undue reservation.
